# Inhibition of sodium-calcium exchanger by tricyclic antidepressants

**DOI:** 10.3389/fphar.2026.1849383

**Published:** 2026-08-17

**Authors:** Sergei I. Boikov, Tatiana V. Karelina, Maxim V. Nikolaev, Dmitry A. Sibarov

**Affiliations:** Sechenov Institute of Evolutionary Physiology and Biochemistry of the Russian Academy of Sciences, Saint-Petersburg, Russia

**Keywords:** amitriptyline, clomipramine, desipramine, neurons, sodium-calcium exchanger, tricyclic antidepressants

## Abstract

**Background:**

Tricyclic antidepressants (TCAs) such as amitriptyline, desipramine, and clomipramine are widely used for the treatment of neuropathic pain, yet the cellular mechanisms underlying their analgesic effects remain incompletely understood. Increasing evidence suggests that, in addition to inhibiting monoamine reuptake, TCAs modulate neuronal calcium signaling.

**Methods:**

As the sodiumcalcium exchanger (NCX) plays a key role in regulating intracellular Ca^2+^ dynamics and calcium-dependent desensitization of NMDA receptors, we investigated whether TCAs directly affect NCX activity. NCX transport currents were recorded using whole-cell patch-clamp in cultured rat cortical neurons, and Ca^2+^ imaging was performed in HEK293 cells expressing NCX1 to assess Ca^2+^ extrusion.

**Results:**

Amitriptyline (10 μM) demonstrated the most prominent effect and significantly inhibited NCX-mediated currents by approximately 80% of the maximal block by Ni^2+^. The magnitude of inhibition was comparable to established NCX inhibitors, including KB-R7943 and SEA0400. Ca^2+^ imaging experiments demonstrated that amitriptyline slowed cytosolic Ca^2+^ clearance in NCX1-expressing cells, indicating inhibition of the forward transport mode responsible for Ca^2+^ extrusion.

**Conclusions:**

These results provide direct evidence that amitriptyline inhibits NCX activity and alters neuronal Ca^2+^ handling. This effect may contribute to enhanced Ca^2+^-dependent desensitization of NMDA receptors and suppression of excitatory signaling, providing a mechanistic explanation of its analgetic properties.

## 1 Introduction

Neuropathic pain is a persistent clinical condition driven by maladaptive synaptic plasticity and sustained neuronal hyperexcitability within peripheral and central nociceptive pathways. Tricyclic antidepressants (TCAs), including amitriptyline, desipramine, and clomipramine, are widely used as first-line agents for neuropathic pain treatment (Moore et al., 2015; Mu et al., 2017; Bates et al., 2019). Their analgesic efficacy occurs at lower doses and with a faster onset than their antidepressant effects, suggesting that mechanisms beyond monoamine reuptake inhibition play a role in pain modulation (Sindrup and Jensen, 1999; Finnerup et al., 2015).

A central determinant of nociceptive signaling and central sensitization is intracellular calcium (Ca^2+^) homeostasis (Prado et al., 2001; Huang et al., 2019; Zhang et al., 2025). Excessive Ca^2+^ influx, particularly through *N*-methyl-D-aspartate receptors (NMDARs), promotes excitotoxicity, oxidative stress and metabolic dysfunction that sustain chronic pain states (Tong et al., 1995). Calcium-dependent desensitization (CDD) of NMDARs serves as an intrinsic protective mechanism limiting Ca^2+^ overload. Enhancement of this process reduces excitatory transmission and is supposed to attenuate pathological pain signaling (Tong et al., 1995; Boikov et al., 2022; Sibarov et al., 2015; Andreeva-Gateva et al., 2024; Stepanenko et al., 2019).

The sodium–calcium exchanger (NCX) is a major regulator of neuronal Ca^2+^ extrusion (Huang et al., 2019), particularly within synaptic microdomains adjacent to NMDARs (Marques-da-Silva, Gutierrez-Merino, 2014). NCX isoforms, primarily NCX1 and NCX3 in the nervous system, shape local Ca^2+^ dynamics following synaptic activity and thereby modulate NMDAR function (Blaustein and Lederer, 1999; Quednau et al., 1997). Inhibition of NCX elevates local intracellular Ca^2+^, potentiating NMDAR CDD and reducing receptor open probability, a mechanism critically dependent on membrane microdomain organization and cholesterol content (Sibarov et al., 2018). Therefore, dysfunction of NCX activity has been implicated in pathological pain states characterized by prolonged Ca^2+^ signaling and enhanced neuronal excitability (Quednau et al., 1997).

Tricyclic antidepressants (TCAs) have been hypothesized to directly inhibit NCX at clinically relevant micromolar concentrations (Stepanenko et al., 2019; Stepanenko et al., 2022; Boikov et al., 2024). Early studies demonstrated that amitriptyline, desipramine and clomipramine suppress Na^+^-dependent Ca^2+^ transport in rat brain synaptosomes, independently of voltage-gated Ca^2+^ channels (Lavoie et al., 1990; Yaksh, 2006). More recent work has established a functional link between TCA-mediated NCX inhibition and enhanced Ca^2+^-dependent suppression of NMDAR currents (Boikov et al., 2022; Stepanenko et al., 2022). Amitriptyline and desipramine enhance NCX1-dependent NMDAR desensitization, an effect abolished by the selective NCX inhibitor SEA0400, whereas clomipramine lacks this Ca^2+^-dependent component, indicating structure-specific NCX engagement (Boikov et al., 2024).

Together, these findings identify NCX as a potential convergent molecular target linking TCA pharmacology, Ca^2+^ homeostasis and suppression of excitatory nociceptive transmission. Here by patch-clamp recording of NCX transport currents and calcium imaging in cultured rat cortical neurons we studied the direct inhibition of NCX by amitriptyline, desipramine, and clomipramine as compared to specific NCX inhibitors — nickel, SEA0400, and KB-R7943.

## 2 Methods

### 2.1 Animals

All animal experiments were carried out on Wistar rats provided by the Sechenov Institute Animal Facility in accordance with the Federation for Laboratory Animal Science Associations (FELASA) criteria and were approved by the Sechenov Institute’s Animal Care and Use Committee (Protocol 1-6/2022 from 21.01.2022). Rats were kept two to four per cage at an ambient temperature of 22 °C–25 °C with a 12-h day/night cycle and free access to water and food. Overall, embryos of fifteen pregnant rats were obtained for the preparation of primary cultures of cortical neurons (about two coverslips with culture per embryo).

### 2.2 Preparation of primary culture of cortical neurons

The 17-day pregnant rats were sacrificed by inhaling CO_2_ for 1 min in a plastic container attached to a CO_2_ tank. Fetal brains were used to make primary cultures of rat cortical neurons, as previously described (Mironova et al., 2007). Neurons were cultured on 7 mm glass coverslips coated with poly-D-lysine in a “Neurobasal” culture medium supplemented with B-27 (ThermoFisher Scientific, Waltham, MA, United States). Experiments were performed after 10–14 days in culture (Mironova et al., 2007; Han and Stevens, 2009).

### 2.3 Patch-clamp recording

Whole-cell currents were recorded from cultured rat cortical neurons using a MultiClamp 700 B patch-clamp amplifier. Data were recorded at 1000 samples/s using Digidata 1440 A controlled by pClamp v10.6 software (Molecular Devices). Solution exchange was performed by means of a fast solution application system, as described earlier (Sibarov et al., 2015).

External bathing solution (BS) contained (in mM): 144 NaCl; 2.8 KCl; 10 HEPES-Na; 2.0 CaCl_2_; 2.0 MgCl_2_ at pH 7.2–7.4, osmolarity 310 mOsm. To suppress the membrane conductance other than NCX, we used the second external bathing solution (BS2) with cocktail of blockers contained (in mM): 144 NaCl; 2.8 KCl; 10 HEPES-Na; 2.0 CaCl_2_; 2.0 MgCl_2_; 10 TEA; 2 CsCl; 0.05 AP5; 0.01 Nifedipine; 0.0001 TTX; 0.001 ouabain. Nifedipine 10 μM and tetrodotoxin 100 nM were added to the extracellular solution to block calcium and sodium voltage-sensitive channels; tetraethylammonium 10 mM and CsCl 2 mM to block potassium voltage-sensitive channels; AP5 (amino-5-phosphonovaleric acid) 50 µM to inhibit NMDA receptors; ouabain 1 µM to block sodium-potassium pump. The pipette solution was composed of (in mM): 120 CsF, 10 CsCl, 10 EGTA, 2 ATP-Mg, and 10 HEPES, osmolarity 300 mOsm, with pH adjusted to 7.4 with CsOH (Cesium based solution was used to block unnecessary potassium conductance).

Patch pipettes of 4–6 MΩ were pulled from RWD B-15086-10 F borosilicate capillaries with filament. Experiments were performed at 22 °C–25 °C. Under control conditions, neurons were voltage clamped at −70 mV. Data are reported without corrections for liquid junction potential, which was measured as −11 mV (Neher, 1992).

**FIGURE 1:**
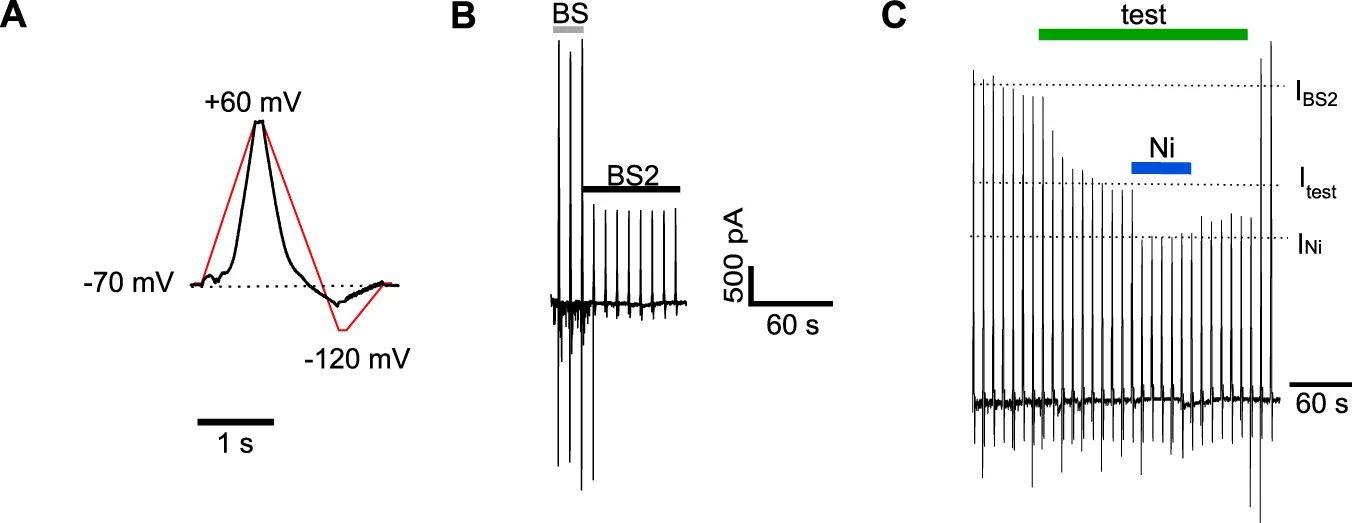
The protocol for recording of NCX transport currents in neurons in primary culture. **(A)** The test voltage applied to the neuron during a single measurement cycle (red) and the sample corresponding current response (black). **(B)** The sample trace recorded in the neuron during periodic application of the test voltage (every 10 s). Elimination of non-NCX currents by when switching from BS to BS2 (see bars above traces), containing the cocktail of inhibitors. **(C)** The recording protocol used in experiments. Bars above recordings indicate application of test substance (test) and 5 mM nickel ions (Ni).

To obtain current–to–voltage (I–V) relations of the cell membranes voltage ramps (Figure 1A) with a speed of ±180 mV s^−1^ ranging from −120 mV to +60 mV were applied at 0.1 Hz as previously reported (Boikov et al., 2022a). Patch was obtained in BS. Measurement of currents was performed in BS2 to avoid the contribution of active conductances other than NCX (Figure 1B).

### 2.4 Analysis of membrane currents

In the presence of BS2, the inhibition of NCX current was evaluated as a fraction of inhibition of current caused by nickel (Egger et al., 1999). Particularly the fraction of inhibition by test substance ‘t' (Figure 1C) was f = (I_BS2_ - I_t_)/(I_BS2_ - I_Ni_). All values are expressed as means ± S.E.M, n indicates the number of experiments. One recording from a single cell on one coverslip was counted as one experiment. Statistical significance of inhibition was evaluated by the one-sample t-test (difference from 0). Experimental groups were compared using ANOVA with Dunnett *post hoc* analysis. *, **, ***, **** - the data are significantly different from zero. *p < 0.05, **p < 0.01, ***p < 0.001, ****p < 0.0001.

### 2.5 Optical detection of calcium

Human embryonic kidney (HEK) 293 T cells were plated onto 15 mm glass coverslips treated with poly-L-lysine (0.2 mg/ml) in 35 mm culture dishes at 1.8–2.5 × 10^5^ cells per glass. Eighteen to twenty-four hours after plating, cells were transiently transfected with Rat NCX1.1 (pcDNA3.1) expression vector (Addgene, Watertown, MA, cat. 75190) using GenJect-39 reagent. Briefly, transfection was performed by adding to each dish 50 µl serum-free medium containing 1 µg total DNA and 2 µl GenJect-39 per 1 mL medium.

48 h after transfection, HEK293 cells were loaded with the Ca^2+^-sensitive fluorescent dye Fluo-8 by incubation in a basic extracellular solution (in mM: 144 NaCl, 2.8 KCl, 2 CaCl_2_, 10 HEPES; pH 7.4; osmolarity 310 mOsm) containing 2 µM Fluo-8 acetoxymethyl ester (Fluo-8 AM; Abcam, Cambridge, United Kingdom) for 60 min at room temperature. Following dye loading, cells were washed for 20 min with dye-free basic solution. Coverslips were then transferred to the stage of a Leica SP5 MP inverted confocal microscope (Leica Microsystems GmbH, Wetzlar, Germany) and continuously perfused with basic solution at a flow rate of 1.2 mL/min.

Drugs were applied using a fast perfusion system, allowing rapid solution exchange. Fluo-8 fluorescence was excited at 488 nm, and emission was collected in the 510–560 nm range. Time-lapse images were acquired at approximately 660-ms intervals (1024 × 1024 pixels; 1.5 frames/s) using a ×20 oil-immersion objective, yielding a field of view of 770 × 770 µm. Image acquisition was performed using Leica LAS AF software v2.3.6 (Leica Microsystems GmbH, Wetzlar, Germany). All experiments were conducted at 24 °C–26 °C.

### 2.6 Analysis of fluorescence

For imaging analysis, the measurements of fluorescence intensity changes over time were made in regions of interest (ROIs) chosen inside HEK293 cells responding to KCl. Measurements were performed using Leica LAS AF software. The average fluorescence intensity of cells (ROIs) during KCl application was considered F_max_. The average fluorescence intensity during the last 30 s of a 1-min washout with BS2 or BS2+ATL was considered F. Mean F/F_max_ from one coverslip was taken as one experiment. Two groups of measurements were compared using Student’s t-test.

### 2.7 Reagents

Most compounds were acquired from Sigma-Aldrich, St. Louis, MO, United States. Particularly CsF cat. 255718; CsCl cat. C4036; CsOH cat. 232041; EGTA cat. 324626; HEPES cat. 54457; ATP-Na cat. A9187; NaCl cat. S9888; KCl cat. P3911; poly-D-lysine cat. P1024; Amitriptyline Hydrochloride cat. A8404; Desipramine Hydrochloride cat. D3900; Memantine Hydrochloride cat. M9292. From VWR (VWR Chemicals, Radnor, PA): HEPES-Na cat.0485; From Tocris (Tocris Bioscience, Minneapolis, MN): KB-R7943 cat. 4144; SEA0400 cat. 6164/10; Clomipramine Hydrochloride cat. 17321-77-6. Neurobasal medium cat. 21103049 and B-27 supplement cat. A3582801 were from ThermoFisher Scientific (Waltham, MA). Lithium Hydrochloride, Nickel Chloride were from RosMedBio (Saint-Petersburg, Russia).

## 3 Results

### 3.1 Electrophysiological analysis of NCX transport currents

Following (Tanaka et al., 2002), a cocktail of ion channel blockers was used to isolate sodium–calcium exchanger (NCX) transport currents and to plot I-V relationships for the tested compounds (Kimura et al., 1999; Boikov et al., 2022a). Experiments were performed in primary cultures of rat cortical neurons, which natively express the NCX1 and NCX3 isoforms (Pignataro et al., 2004). To suppress conductances and electrogenic events unrelated to NCX (Figure 1B), blockers of voltage-gated K^+^, Na^+^, and Ca^2+^ channels, as well as NMDA receptors and Na/K-ATPase, were included in the perfusion solution.

**FIGURE 2:**
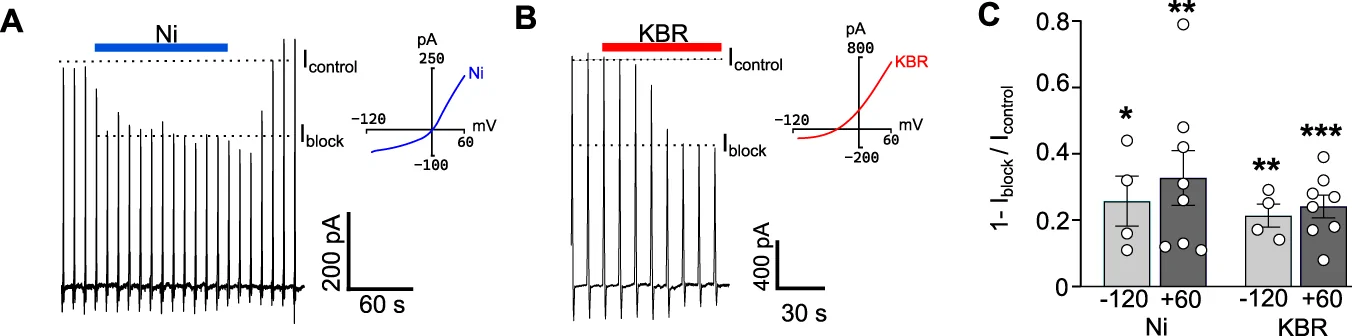
NCX transport current inhibition by common blockers in primary cortical neuronal culture. The concentrations of calcium [Ca^2+^] ions in BS2 solution are 2 mM. **(A)** Examples of transmembrane currents when nickel chloride was applied at a concentration of 5 mM (Ni). The insert at right illustrates the current-to-voltage plot of NCX current blocked by Ni^2+^. **(B)** Examples of transmembrane currents when KB-R7943 was applied at a concentration of 10 μM (KBR). The insert at right illustrates the current-to-voltage plot of NCX current blocked by KBR. **(C)** The histogram represents the fractions of blocked current in forward and in reverse mode by nickel and KB-R7943 (1 – I_block_/I_control_, where I_block_ is current in the presence of a blocker and I_control_ obtained in the BS2). Data from each experiment (symbols) and mean values ± S.E.M. are shown. *, **, *** - the data are significantly different from zero. Mean fractions of blocked current values are summarized in Table 1.

The addition of the blocker cocktail reduced nonspecific membrane conductance, resulting in a decrease of current to 57% ± 3% of control (100% * I_bs2_/I_bs_; n = 20; Figure 1B). All subsequent experiments were therefore performed in the BS2 basic solution. As reference inhibitors providing complete NCX block, 5 mM nickel chloride (Iwamoto et al., 1999) or 10 μM kB-R7943 (Iwamoto et al., 1996) were used. Both nickel ions (Figure 2A) and KB-R7943 (Figure 2B) inhibited NCX transport currents with comparable efficacy in the forward (NCX_forw_) and reverse (NCX_rev_) operating modes of the exchanger (Figure 2C). The I-V characteristics of blocked NCX current (inserts at Figures 2A,B) show measurable NCX_forw_ and NCX_rev_ currents at negative and positive membrane voltages respectively. The degree of trans-membrane current inhibition did not differ between the two inhibitors in either mode (unpaired t-test, n = 8, p > 0.8) and was approximately 30% (Table 1). For both exchanger modes of operation (NCX_forw_ and NCX_rev_) stable inhibition was achieved in 40–60 s and was significant for both nickel and KB-R7943 (Figure 2C; Table 1).

**TABLE 1:** The fractions of blocked current values in forward (forw) and in reverse (rev) modes by nickel (Ni) and KB-R7943 (KBR) in neurons.

Blocked current	Ni_rev_	Ni_forw_	KBR_rev_	KBR_forw_
1 - I_block_/I_control_	0.33 ± 0.09 (n = 8) **, p = 0.0054	0.26 ± 0.09 (n = 4) *, p = 0.0422	0.24 ± 0.04 (n = 8) ***, p = 0.0002	0.21 ± 0.04 (n = 4) **, p = 0.0088

*, **, *** - the data are significantly different from zero indicating the presence of the effect.

Based on the observation that amitriptyline, and desipramine have a calcium-dependent effect on NMDA receptors (Stepanenko et al., 2019, 2022; Boikov et al., 2024) similar to the effect of NCX inhibitor KB-R7943 (Sibarov et al., 2015), we suggested that the tricyclic antidepressants (TCAs) under study act on NCX. For comparison, we also analyzed three additional compounds that exhibit a calcium-dependent effect on NMDA receptors and inhibit NCX: memantine (Glasgow et al., 2017; Brittain et al., 2012), lithium, and SEA0400 (Boikov et al., 2022; Sibarov et al., 2015; Boikov et al., 2024). Since data on the effective concentration of SEA0400 required to inhibit the exchanger vary depending on the cell type (Matsuda et al., 2001), we used it at two concentrations: 5 and 50 nM. Memantine was used at a concentration of 10 μM, similar to TCAs. Lithium ions, as a non-transportable substrate for the exchanger, were used at a concentration of 1.8 mM, as this concentration is sufficient to elicit the calcium-dependent effects of lithium on NMDA receptors (Boikov et al., 2022).

**FIGURE 3:**
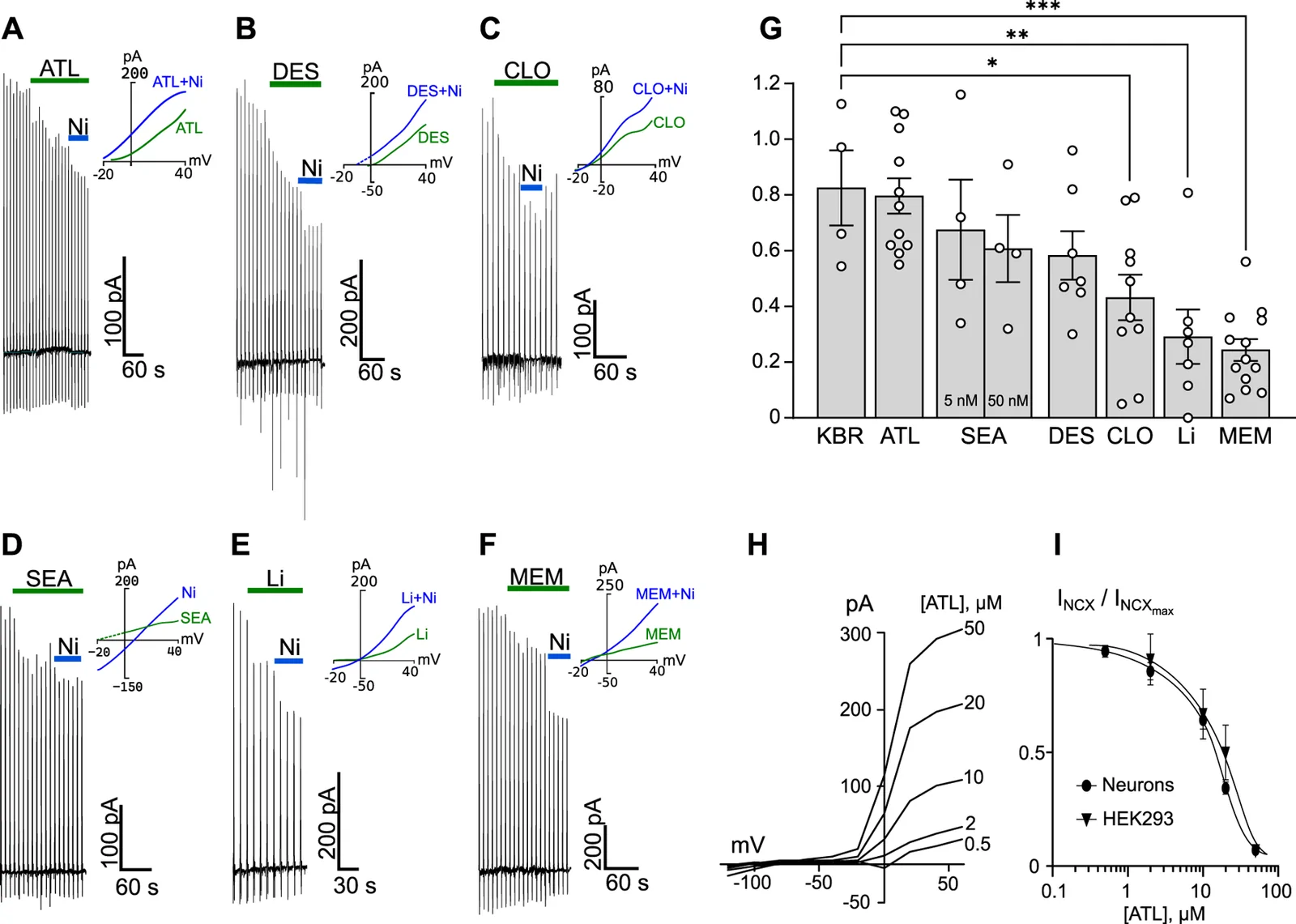
Effect of tricyclic antidepressants and NCX modulators on transmembrane currents in primary cortical neuronal culture. The concentration of calcium [Ca^2+^] ions in the BS2 solution is 2 mM. Bars above traces indicate applications of test substances (ATL, DES, CLO, SEA, Li, MEM) and 5 mM nickel ions (Ni) to obtain full inhibition of the exchanger. Panels **(A–F)** illustrate the effects of test substances on NCX transmembrane currents. The insert at right illustrates the current-to-voltage plot of NCX current blocked by each of the test substances and substances in the presence of Ni^2+^. **(A)** 10 μM amitriptyline (ATL), **(B)** 10 μM desipramine (DES), **(C)** 10 μM clomipramine (CLO), **(D)** 50 nM SEA0400 (SEA), **(E)** 1.8 mM lithium (Li), **(F)** 10 µM memantine (MEM) correspondently. **(G)** The histogram represents the fractions of NCX_rev_ inhibited current by test substances relative to full block by Ni^2+^. **(H)** Sample current-to-voltage plots of the amitriptyline-blocked current obtained from a neuron in the presence of various ATL concentrations. **(I)** An average concentration-inhibition relationship for the ATL effect on NCX currents obtained for neurons (n = 4) and HEK293 cells expressing NCX1 (n = 4). Symbols show mean values ± S.E.M. of the relative amplitudes of NCX currents obtained at different ATL concentrations. The solid line is the fit to the data with the Hill equation. Data from each experiment (symbols) and mean values ±S.E.M. are shown. *, **, *** - the data are significantly different from KB-R7943. Mean fractions of blocked current values are summarized in Table 2.

All tested compounds—namely, KB-R7943, amitriptyline, desipramine, clomipramine, memantine, lithium, and SEA0400—significantly inhibited the exchanger current in both reverse (at V_m_ = +60 mV) and forward (at V_m_ = −120 mV) operating modes (Figures 3A–F; Table 2). Under our experimental conditions, the exchanger operates predominantly in the reverse mode, yielding a substantially larger absolute transport current for NCX_rev_ compared to NCX_forw_ (Figures 3A–F). Therefore, we ranked the compounds by relative blocking efficacy based on the more precisely measured NCX_rev_ (Figure 3G; Table 2). This approach is justified, as the blocking efficacy of known exchanger inhibitors does not significantly differ between NCX_rev_ and NCX_forw_ (Tanaka et al., 2002).

**TABLE 2:** NCX transport current inhibition by test substances presented as a fraction of full inhibition produced by nickel. Shapiro-Wilk test was used for normality verification. Difference from 0 by one-sample t-test indicates significant NCX inhibition. Difference from KB-R7943 was established by one-way ANOVA with Dunnett multiple comparison test.

Substance	Mean ± SEM	n	Normality	Differ from 0	Differ from KB-R7943
Reverse mode block at V_m_ = +60 mV					
KB-R7943 10 μM	0.74 ± 0.10	8	yes	***, p = 0,0002	​
Amitriptyline 10 μM	0.80 ± 0.06	11	yes	****, p=<0,0001	​
Sea 5 nM	0.68 ± 0.18	4	yes	*, p = 0.033	​
Sea 50 nM	0.61 ± 0.12	4	yes	*, p = 0,0151	​
Desipramine 10 μM	0.58 ± 0.09	7	yes	***, p = 0,0005	​
Clomipramine 10 μM	0.43 ± 0.08	10	yes	***, p = 0,0005	*, p = 0,0279
Lithium 1.8 mM	0.29 ± 0.09	7	yes	*, p = 0.046	**, p = 0.003
Memantine 10 μM	0.24 ± 0.04	13	yes	****, p=<0,0001	***, p = 0,0003

Substance	Mean ± SEM	n	Normality	Differ from 0
Forward mode block at V_m_ = −120 mV				
KB-R7943 10 μM	0.82 ± 0.13	4	yes	**, p = 0,0088
Amitriptyline 10 μM	0.41 ± 0.06	11	yes	*, p = 0.045
Sea 5 nM	0.43 ± 0.07	3	yes	*, p = 0,0381
Sea 50 nM	0.35 ± 0.08	4	yes	​
Desipramine 10 μM	0.18 ± 0.07	6	yes	​
Clomipramine 10 μM	0.45 ± 0.21	9	yes	​
Lithium 1.8 mM	0.21 ± 0.09	8	yes	​
Memantine 10 μM	0.31 ± 0.08	9	yes	​

*, **, *** - the data are significantly different.

A comparison of amitriptyline, desipramine, clomipramine, memantine, lithium, and SEA0400 with KB-R7943 (one-way ANOVA, Table 2) revealed that the non-selective blocker KB-R7943, the selective NCX1 blocker SEA0400, and the tricyclic antidepressants amitriptyline and desipramine exhibited similar blocking efficacy. In contrast, clomipramine, lithium, and memantine were significantly less potent than KB-R7943 (Table 2). These findings are consistent with earlier observations that, in neurons where NCX is not blocked, amitriptyline and desipramine induce calcium-dependent effects on NMDA receptors (Stepanenko et al., 2019, 2022), whereas clomipramine (Stepanenko et al., 2022) and the classical NMDA receptor channel blocker memantine (Glasgow et al., 2017) do not show noticeable calcium dependence in their actions.

As shown, among the compounds tested, amitriptyline exhibited the highest efficacy in blocking the exchanger, on par with KB-R7943. Notably, amitriptyline is the most frequently cited and recommended TCA for neuropathic pain (Moore et al., 2015), while NCX inhibition—specifically via KB-R7943—has been demonstrated to be effective in treating neuropathic pain in animal models (Huang et al., 2019; Andreeva-Gateva et al., 2024). Accordingly, we aimed to determine the effective concentration of amitriptyline required to elicit NCX block.

For the experimental conditions used in this study, we estimated the half-effective concentration of amitriptyline-induced exchanger current inhibition. For this purpose, the current-to-voltage characteristics of the amitriptyline-blocked current were plotted in the presence of various amitriptyline concentrations (Figure 3H). At V_m_ = +50 mV, we plotted the concentration curves of NCX_rev_ inhibition for neurons and HEK293 cells (Figure 3I). For NCX natively expressed by cortical neurons, the IC_50_ was 10.7 ± 3.8 µM (n = 4). For NCX1 isoform expressed in HEK293 cells, we obtained a similar IC_50_ value of 15 ± 1.6 µM (n = 4). The almost identical IC_50_ value for neurons expressing mixed NCX isoforms, as well as for HEK293 expressing only NCX1, suggests that NCX1 is the main target for amitriptyline action in neurons.

The design of our experiments allowed us to primarily record NCX_rev_ current, while the amplitude of the NCX_forw_ current was small. This limited the reliability of the results on the block of NCX_forw_ by the studied substances.

### 3.2 Optical detection of Ca^2+^ clearance by NCX_forw_

**FIGURE 4:**
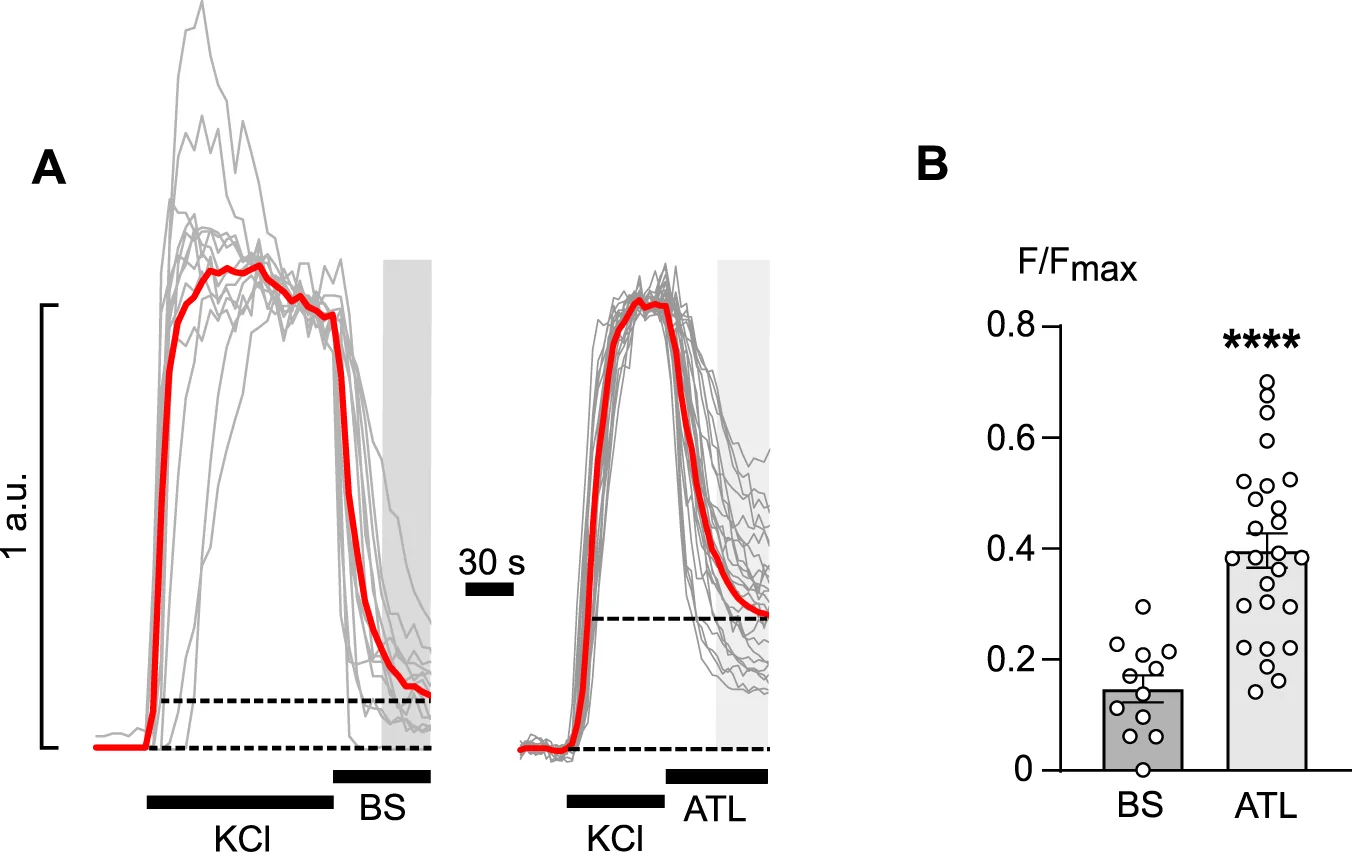
The decay of KCl-induced Fluo-8 fluorescence during washout with basic solution (BS) or basic solution containing 10 µM amitriptyline (ATL) in HEK293 cells expressing NCX1. **(A)** Sample traces of cells fluorescence (gray lines) and average response (red line) from one experiment. The grey bar indicated a 30 s interval where the integral of fluorescence was calculated. **(B)** Integrals of fluorescence during washout with BS or ATL (****, p < 0.0001, unpaired t-test) reveal a slowing down of calcium extrusion in the presence of amitriptyline. Circles indicate individual experiments (BS, n = 12; ATL, n = 26).

To overcome this limitation, additional experiments were conducted to evaluate the effect of amitriptyline on the functioning of the exchanger in forward mode (NCX_forw_). In HEK293 cells expressing NCX1 and loaded with the fluorescent Ca^2+^ sensitive dye Fluo-8, 70 mM KCl was applied to induce NCX_rev_-mediated Ca^2+^ entry into the cytoplasm. Forced depolarization of the cell membrane with KCl induced calcium responses only in a fraction of cells in the NCX1-transfected culture. In the untransfected cultures, calcium responses to KCl were absent, indicating calcium entry into the cells only through a functional exchanger during depolarization. It should also be noted that in the presence of 10 μM amitriptyline, KCl did not evoke any calcium responses (n = 5, p = 0.94, one-sample t-test), which confirms the electrophysiological data on amitriptyline inhibition of NCX_rev_. Then during washout we observed a gradual decrease of Fluo-8 fluorescence (n = 12), reflecting the process of Ca^2+^ removal from the cytoplasm by NCX_forw_ (Figure 4A). This approach of analyzing the descending phase of the calcium response to assess the block of the NCX_forw_ was used earlier in the work by Namekata (Namekata et al., 2006). We compared the integral of Fluo-8 fluorescence during last 30 s of 1 min washout with a basic solution and one containing amitriptyline (10 µM, n = 26) (Figure 4A). The calcium signal during washout in the presence of amitriptyline was larger than in control (Figure 4B) indicating a slowing down of calcium extrusion by NCX_forw_ by amitriptyline. Although these data do not allow us to assess the degree of exchanger inhibition by amitriptyline, they are sufficient to attest the reliability of this effect.

## 4 Discussion

In this study, we provide direct electrophysiological and imaging evidence that some TCAs inhibit the neuronal sodium–calcium exchanger. Using isolated NCX transport currents in cultured rat cortical neurons and complementary Ca^2+^ imaging in NCX1-expressing HEK293 cells, we demonstrate that amitriptyline and desipramine substantially suppress NCX activity. It especially concerns amitriptyline, whose IC_50_ for NCX inhibition of ∼10 µM was not substantially different from those of most commonly used NCX inhibitor KB-R7943 (∼5–7 µM) (Iwamoto and Shigekawa, 1998; Tanaka et al., 2002). Therefore, we selected KB-R7943 as a reference isoform non specific NCX blocker. This choice was primarily driven by its extensive characterization, covering both its direct effects on NCX and its off-target effects on NMDA receptors and mitochondrial respiration (Brustovetsky et al., 2010), as well as voltage-gated calcium and potassium channels (Watano et al., 1996). In our experiments, we avoided interference from these off-target effects by using a cocktail of blockers. Specifically, during the measurement of the exchanger transport current, all non-NCX sodium, calcium, and potassium conductances were blocked, thereby preventing them from distorting our measurements. Consequently, the ∼74% inhibition of the reverse NCX current (NCX_rev_) by 10 µM KBR observed in our experiments is in complete agreement with the ∼80% blocking reported by other authors (Amran et al., 2003; Brustovetsky et al., 2010).

Our objective was not to construct detailed concentration–response curves for each compound, but to compare their relative ability to inhibit NCX as compared to selective NCX blockers. Therefore, a selected concentration of TCAs of 10 µM is a commonly used reference concentration for NCX inhibitors such as KB-R7943, SN6, etc. (Iwamoto et al., 1996; Niu et al., 2007). IC_50_ values for NCX inhibition vary substantially depending on exchanger isoform, membrane potential, ionic gradients, and cell type and are therefore not directly transferable to our preparation. Additionally, the selected concentration lies within the micromolar range previously shown to modulate NCX and Ca^2+^-dependent NMDAR CDD in neurons.

Patch-clamp recordings revealed that all three TCAs significantly inhibited NCX-mediated currents, with the most reliable measurements obtained for the reverse mode of operation (NCX_rev_). The almost identical IC_50_ value for neurons expressing mixed NCX isoforms, as well as for HEK293 expressing only NCX1, suggests that NCX1 is the main target for amitriptyline action in neurons. A complementary study of calcium accumulation/removal in NCX1-expressing HEK293 cells showed that amitriptyline inhibited both calcium entry into the cell via the NCX_rev_ exchanger and calcium removal from the cell via the NCX_forw_ exchanger.

In patch-clamp experiments on neurons, amitriptyline produced the most pronounced suppression of both NCX_rev_ and NCX_forw_ currents on par with KB-R7943, while clomipramine caused twice-weaker inhibition. In the treatment of neuropathic pain, the analgesic effect of clomipramine is primarily attributed to its inhibition of serotonin reuptake (Farghaly et al., 2012). However, because clomipramine has only a weak affinity for the sodium-calcium exchanger (NCX), it does not affect NMDA receptor (NMDAR) desensitization at clinically relevant concentrations (Stepanenko et al., 2022).

In contrast, amitriptyline significantly enhances calcium-dependent NMDAR desensitization at therapeutic concentrations (Stepanenko et al., 2019). Notably, this effect is conditional: it requires both a functionally active NCX (Stepanenko et al., 2019) and the co-localization of NCX and NMDARs within lipid membrane microdomains (Boikov et al., 2022). The most plausible mechanism is that amitriptyline-induced inhibition of NCX leads to a localized accumulation of calcium within these microdomains, which in turn potentiates NMDAR desensitization. Given that NMDAR blockade has long been recognized as an effective strategy for managing neuropathic pain (Aiyer et al., 2018), this off-target effect of amitriptyline on NMDARs—mediated via NCX inhibition—likely contributes to its overall analgesic efficacy. This mechanism is independent of, and complementary to, its classic monoamine reuptake inhibition.

Supporting this hypothesis, the selective NCX inhibitor KB-R7943, which has no effect on monoamine reuptake, also demonstrates analgesic properties in neuropathic pain models (Andreeva-;Gateva et al., 2024). This provides indirect evidence that NCX inhibition represents a relevant and potentially efficacious therapeutic target for neuropathic pain.

In our study, we also examined two additional compounds that inhibit the sodium–calcium exchanger (NCX): lithium (Ander et al., 2007; Boikov et al., 2022a) and memantine (Brittain et al., 2012). Both agents inhibited the exchanger approximately 2.5 times more weakly than amitriptyline. Nevertheless, only memantine has shown clinical promise, particularly for chemotherapy-induced peripheral neuropathy (Esmaili et al., 2026), although the overall evidence for its efficacy across all neuropathic pain conditions remains mixed and insufficient to support its use as a first-line therapy. Notably, memantine’s primary mechanism is likely related to direct channel block of NMDA receptors, which occurs at lower concentrations than those required for its calcium-dependent effects (Glasgow et al., 2017). In contrast, lithium is not a standard treatment for neuropathic pain.

When comparing the amitriptyline concentrations used in this study with those in patient tissues during the treatment of neuropathic pain, one cannot limit oneself to the accumulation of this drug in the cerebrospinal fluid. On the one hand, 0.5 µM amitriptyline in CSF corresponds to an overdose in humans (Hultén et al., 1992), which is significantly lower than the IC_50_ for inhibition of NCX. On the other hand, amitriptyline can accumulate in the brain, and its concentration in the brain after crossing the blood-brain barrier is 1500 ng/g – 2000 ng/g wet brain tissue (Amin and Bano, 2014), which corresponds to 5–7 µM enough for substantial NCX inhibition. The local drug accumulation in membranes or intracellular compartments (Jeanson et al., 2016; Jansen et al., 2019) may permit higher effective concentrations at specific tissues.

In summary, our results demonstrate that amitriptyline directly inhibit NCX activity in both neuronal and heterologous systems. By altering NCX-dependent Ca^2+^ handling, amitriptyline can modify intracellular Ca^2+^ dynamics and therefore enhance Ca^2+^-dependent NMDAR desensitization, which provides a mechanistic basis for their effects on excitatory Ca^2+^ signaling.

## Data Availability

The original contributions presented in the study are included in the article/supplementary material, further inquiries can be directed to the corresponding author.
